# Sexual Function and Psychological Distress Across Surveillance, Colposcopy, and LEEP in Women with a History of High-Risk HPV Positivity: A Cross-Sectional Study

**DOI:** 10.3390/jcm15197386

**Published:** 2026-09-23

**Authors:** Basak Ozge Kayan, Gokce Nur Esen Topal

**Affiliations:** Department of Obstetrics and Gynecology, Division of Gynecologic Oncology, Basaksehir Cam and Sakura City Hospital, Istanbul 34480, Türkiye; gokcenuresentopal@gmail.com

**Keywords:** human papillomavirus, cervical dysplasia, colposcopy, LEEP, sexual dysfunction, quality of life, depression

## Abstract

**Background/Objectives:** High-risk human papillomavirus (hrHPV) positivity and its management may affect sexual function and psychological well-being, yet whether this burden differs across the successive steps of cervical management is unclear. We compared sexual function, quality of life, and psychological distress across three management steps—surveillance, colposcopy, and loop electrosurgical excision procedure (LEEP). **Methods:** In this single-center cross-sectional study, 302 reproductive-age women with a history of hrHPV positivity (surveillance, n = 50; colposcopy, n = 177; LEEP, n = 75) completed the Female Sexual Function Index (FSFI), WHOQOL-BREF, and Hospital Anxiety and Depression Scale (HADS). Groups were compared with Kruskal–Wallis and chi-square tests; FSFI total was modelled by multivariable linear regression and FSFI-defined dysfunction by logistic regression. **Results:** No statistically significant between-group differences were observed in FSFI total (medians 23.8/25.2/24.5; *p* = 0.897), FSFI subdomains, HADS, or WHOQOL domains; FSFI-defined dysfunction was frequent across groups, with no statistically significant between-group difference (59–67%). No statistically significant association between management pathway and FSFI was observed in the adjusted models (LEEP vs. surveillance B = −0.63, 95% CI −4.68 to +3.42). Depressive symptoms—but not anxiety—were associated with FSFI-defined dysfunction (per HADS-D point, OR = 1.14, 95% CI 1.03–1.26, *p* = 0.009), whereas the association with continuous FSFI was not significant (B = −0.37, *p* = 0.064). Marital status was the strongest overall correlate. **Conclusions:** Across surveillance, colposcopy, and LEEP, sexual function did not differ significantly between management groups at the time of assessment, and, in exploratory analyses, depressive symptoms showed the most consistent association with sexual dysfunction. These findings highlight the potential relevance of psychosocial and relational context in the psychosexual care of women with a history of hrHPV positivity, although prospective studies are needed before specific screening or counselling recommendations can be made.

## 1. Introduction

Cervical cancer remains the fifth most commonly diagnosed cancer and the fourth leading cause of cancer death among women worldwide, with an estimated 604,000 new cases and 280,000 deaths in 2024, most of them in low- and middle-income settings [1]. Persistent infection with an oncogenic (high-risk) human papillomavirus (HPV) is the necessary cause of virtually all cervical precancer and cancer, although most infections become undetectable within one to two years [2]. Prevention therefore rests on vaccination and on organised screening with cytology and high-risk HPV (hrHPV) testing, followed, where indicated, by colposcopy and by excisional treatment such as the loop electrosurgical excision procedure (LEEP) [3].

For the individual woman, a positive hrHPV result is a psychosocial event as much as a medical one. More than half of women who learn of an abnormal screening result report distress, and the interval between diagnosis and treatment is often marked by carcinophobia (fear of having or developing cancer), uncertainty, and concern about sexual relationships [4,5]. Beyond fear of malignancy, HPV positivity may also be accompanied by perceived stigma, concerns about disclosure to partners, and reduced sexual self-confidence, extending its impact beyond the clinical setting [4]. Each management step may add its own burden: colposcopy is frequently experienced as an anxiety-laden diagnostic threshold [5], whereas excisional treatment introduces a physical intervention with possible effects on sexual function [6,7]. In routine care these procedures are commonly viewed as technical steps, and women’s psychosocial needs may be overlooked.

Evidence on how these steps affect sexual function is inconsistent. Some studies report lower FSFI scores after colposcopy and LEEP [6,7], whereas several prospective studies find no significant change—or even improvement—after treatment and attribute residual difficulty to cancer-related fear or to the vaginal microenvironment rather than to the excision itself [8,9,10]; cross-sectional findings are similarly mixed [11]. A recurring theme in quality-of-life research is that psychological distress, particularly depressive symptoms, is more strongly associated with impaired quality of life than sexual or procedural factors are [5,12,13]. However, most studies examine a single group, and few have compared surveillance, colposcopy, and LEEP within one cohort using sexual-function, quality-of-life, and mood measures together.

We therefore studied reproductive-age women with a history of hrHPV positivity, grouped by management step. The primary aim was to compare sexual function (FSFI) across the three management pathways. Secondary aims were to compare quality of life (WHOQOL-BREF) and psychological distress (HADS), and to determine whether psychological distress was more strongly associated with sexual function than the management pathway itself.

## 2. Materials and Methods

### 2.1. Study Design and Participants

This single-center cross-sectional study was conducted between 17 June and 31 July 2026 at the gynecologic oncology outpatient clinic of a tertiary referral hospital (Basaksehir Cam and Sakura City Hospital, Istanbul, Turkiye) and is reported in accordance with the STROBE Statement. During the study period, all consecutive eligible women attending the clinic were invited to participate, and those who provided written informed consent were enrolled. No financial or other incentives were offered for participation.

Eligible participants were women aged 18–49 years with a documented history of hrHPV positivity. They were required to have been sexually active during the preceding four weeks and to be able to complete the study questionnaires. Women were excluded if they were menopausal, pregnant, or postpartum; had a gynecologic malignancy, an active psychiatric disorder, chronic pelvic pain, or another systemic condition likely to affect sexual function; or had an active genital infection. Women who had not been sexually active during the preceding four weeks were also excluded.

Participants completed the questionnaires before their clinical consultation, during a routine outpatient visit in a private setting. Participation was voluntary; the decision to participate or decline had no bearing on the patient’s clinical care, and the study did not influence diagnostic evaluation or subsequent management. The study was conducted in accordance with the Declaration of Helsinki and approved by the Clinical Research Ethics Committee No. 1 of Basaksehir Cam and Sakura City Hospital (approval no. E-96317027-514.10-317491029; 16 June 2026).

### 2.2. Clinical Management Pathways

Participants were classified according to the highest level of cervical management received: surveillance, colposcopy, or LEEP [3]. Women in the surveillance group were followed with cervical cytology and hrHPV testing alone, without colposcopy. The colposcopy group underwent colposcopy, with or without biopsy, but no excisional treatment. Women who underwent both colposcopy and LEEP were assigned to the LEEP group. Women in the LEEP group underwent excisional treatment, most commonly after colposcopy and a directed biopsy demonstrating CIN2/CIN3 or persistent CIN1 (per institutional protocol, persistence ≥ 2 years); less commonly, LEEP was performed for cytology–histology discordance or as an expedited (“see-and-treat”) excision without prior colposcopy. Clinical and pathological data, including cervical cytology, colposcopic findings, cervical biopsy results, and LEEP histopathology, were retrieved from the electronic medical records.

Because clinical management was determined by the underlying cervical findings, management pathway and cervical-abnormality severity were closely related and were not treated as independent exposures in the multivariable analysis. Management pathway was retained as the primary exposure, whereas the composite cervical-abnormality severity score was evaluated separately in a sensitivity analysis (Section 2.8). For women who had undergone colposcopy or LEEP, the interval between the procedure and questionnaire completion was not recorded; therefore, time since the procedure could not be incorporated into the analysis.

### 2.3. HPV Testing and Genotype Classification

HPV testing and cervical cytology were performed at the study hospital. For women who had previously undergone HPV or cytology testing at an outside institution, both tests were repeated in-house, and these in-house results were used for classification. Cytological samples were collected using a liquid-based method (ThinPrep Pap test; Hologic, Marlborough, MA, USA). Testing for hrHPV was performed with the cobas 4800 HPV Test (Roche Molecular Systems, Pleasanton, CA, USA), which separately identifies HPV16, HPV18, and a pooled panel of 12 other high-risk types (31, 33, 35, 39, 45, 51, 52, 56, 58, 59, 66, and 68).

Because this assay comprises three channels (HPV16, HPV18, and the pooled other-hrHPV panel), a “multiple” result—positivity in at least two channels—necessarily involves HPV16 and/or HPV18. Genotype was therefore classified into three categories for analysis: HPV16/18-containing (isolated HPV16, isolated HPV18, or any multiple infection), isolated other hrHPV (reference), and currently hrHPV-negative—the last representing women with documented prior hrHPV positivity who were negative on the most recent HPV test at the time of assessment. For descriptive analyses the original five result categories (isolated HPV16, isolated HPV18, multiple infection, isolated other hrHPV, and currently hrHPV-negative) were retained, whereas for the multivariable analyses HPV16, HPV18, and multiple infections were combined into a single HPV16/18-containing category.

### 2.4. Female Sexual Function Index (FSFI)

The FSFI [14] is a 19-item instrument assessing six domains (desire, arousal, lubrication, orgasm, satisfaction, and pain) over the preceding four weeks. Each domain score is the sum of its items multiplied by a domain factor, and the total (range 2–36) is the sum of the six weighted domain scores, with higher scores indicating better function. The validated Turkish version was used [15]. A total score below 26.55 was pre-specified to indicate sexual dysfunction [16].

### 2.5. WHOQOL-BREF

The Turkish WHOQOL-BREF [17] comprises the four standard WHOQOL-BREF domains—physical health, psychological, social relationships, and environment—each transformed to a 0–100 scale, with higher scores indicating better quality of life. The four domains were scored using the standard 26-item WHOQOL-BREF algorithm [18]. The Turkish version includes an additional national item, which was administered but not incorporated into the four standard domain scores, so as to preserve the standard WHOQOL-BREF scoring algorithm and facilitate international comparability. Domain scores were computed after reverse-scoring the relevant items and applying the standard ≥80% item-completeness rule [18].

### 2.6. Hospital Anxiety and Depression Scale (HADS)

The HADS comprises two seven-item subscales assessing anxiety (HADS-A) and depressive symptoms (HADS-D), each scored from 0 to 21, with higher scores indicating greater symptom burden [19,20]. The validated Turkish version was used, and HADS is a screening instrument for symptom severity rather than a diagnostic tool. In the primary analyses, both subscales were treated as continuous variables. The Turkish validation identified screening cut-offs of 10 for HADS-A and 7 for HADS-D; threshold-based (dichotomous) analyses of HADS-D were exploratory and are reported in the Supplementary Materials.

### 2.7. Outcomes and Covariates

The primary outcome was the FSFI total score; secondary outcomes were the six FSFI domain scores, FSFI-defined sexual dysfunction (FSFI total < 26.55), the four WHOQOL-BREF domains, and HADS-A and HADS-D. Covariates were age, marital status, education, smoking, parity, and HPV genotype; marital status was dichotomized as married versus not married for the primary multivariable analyses (a three-category sensitivity analysis is reported in the Supplementary Materials). A composite cervical-abnormality severity score, used only in a sensitivity model, was defined as the highest documented abnormality—the maximum of the cytology-derived grade (0 = normal; 1 = ASC-US/LSIL; 2 = ASC-H/HSIL/AGC/AGUS) and the biopsy-derived grade (0 = normal; 1 = CIN1; 2 = CIN2/CIN3)—with cytology determining the grade when biopsy was not performed.

### 2.8. Statistical Analysis

The sample size was pre-specified in the protocol. Under a one-way analysis-of-variance framework, detecting a medium between-group effect (Cohen’s f ≈ 0.25) in FSFI total with 80% power at a two-sided α = 0.05 required approximately 158 participants; the achieved sample (n = 302) substantially exceeded this.

Continuous variables are summarised as median [interquartile range] and categorical variables as n (%). Because continuous outcomes were left-skewed, the three groups were compared with the Kruskal–Wallis test (Dunn’s post-hoc test with Holm correction was planned only following a significant omnibus test and was therefore not required) and categorical variables with Pearson’s chi-square test, or a Monte Carlo approximation of the Fisher–Freeman–Halton exact test for tables with sparse expected counts. Group effect sizes are reported as epsilon-squared (ε^2^), with negative sample estimates truncated at zero.

The primary endpoint, FSFI total, was modelled by multivariable linear regression with heteroscedasticity-consistent (HC3) standard errors, in three hierarchical models fitted on a common complete-case sample: Model 1 (management group only), Model 2 (+age, marital status, education [categorical], smoking, parity, HPV genotype), and Model 3 (+HADS-A and HADS-D). Because management group and cervical disease severity were structurally confounded—treatment allocation reflected severity, and biopsy was undefined for the surveillance group—the two were not entered into the same model; severity was assessed in a separate sensitivity model in place of group (Sensitivity A), and a further analysis was restricted to women who remained HPV-positive (Sensitivity B). As an additional sensitivity analysis (Sensitivity C), we refitted the primary multivariable FSFI model after restricting the analysis to married women (n = 181), with marital status omitted from the model. In a further sensitivity analysis (Sensitivity D), the primary model was additionally adjusted for BMI among participants with complete BMI and covariate data (n = 132). FSFI-defined dysfunction (<26.55) was analysed by logistic regression as a secondary endpoint.

Model assumptions (normality, heteroscedasticity, multicollinearity, and influence) were examined; because heteroscedasticity was present, HC3-robust standard errors were used (full diagnostics in the Supplementary Materials). Associations among FSFI, HADS, and WHOQOL domains were examined by Spearman correlation (exploratory; Benjamini–Hochberg correction). Analyses were complete-case, with two-sided *p* < 0.05 considered significant. Data were managed in IBM SPSS Statistics v31; statistical analyses were performed in Python 3 using pandas, statsmodels (linear regression with HC3 standard errors and logistic regression by maximum likelihood), SciPy (Kruskal–Wallis and Spearman correlation) with a custom Monte Carlo procedure for the Fisher–Freeman–Halton exact test, and scikit-learn (area under the ROC curve).

Use of generative artificial intelligence: generative AI tools (OpenAI ChatGPT and Anthropic Claude) were used to improve the English language of the manuscript, including grammar, wording, readability, and overall language quality. All data analyses, results, and interpretations were reviewed and revised by the authors, who take full responsibility for the content.

## 3. Results

### 3.1. Participant Characteristics

Among 304 enrolled women, two could not be classified by management pathway because colposcopy/LEEP status was unavailable, leaving 302 for the group-based analyses (surveillance, n = 50; colposcopy, n = 177; LEEP, n = 75; Figure 1). The groups were similar in age, education, smoking, and parity but differed in marital status (*p* = 0.045). HPV genotype, cytology, and biopsy findings differed substantially across the three groups (all *p* < 0.001); CIN2+ on biopsy was present in 0%, 0.6%, and 78.7% of the surveillance, colposcopy, and LEEP groups, respectively (Table 1). Overall, 34 women (11.3%) with documented prior hrHPV positivity were hrHPV-negative at the study visit, most of them in the surveillance group. Because body mass index was available for only about half of the participants, the apparent between-group difference should be interpreted cautiously (Table 1, footnote).

### 3.2. Sexual Function, Quality of Life, and Mood Across Groups

Despite these differences in clinical characteristics, no statistically significant between-group differences were observed for any patient-reported outcome (Table 2). FSFI total scores did not differ significantly (medians 23.8, 25.2, and 24.5; *p* = 0.897) with a negligible effect size (ε^2^ ≈ 0), as were all six FSFI subdomains, HADS-A and HADS-D, and the four WHOQOL-BREF domains (all *p* > 0.05). FSFI-defined sexual dysfunction was frequent across groups, with no statistically significant between-group difference (surveillance 67.3%, colposcopy 59.4%, LEEP 59.5%; *p* = 0.584), and adjusted point estimates for between-group differences in FSFI total remained small (Table 3).

### 3.3. Multivariable Analysis of Sexual Function

In hierarchical linear regression fitted on a common complete-case sample (n = 266), no statistically significant association was observed between the management group (colposcopy or LEEP vs. surveillance) and FSFI total in the unadjusted model, after adjustment for sociodemographic and HPV variables, or after adding psychological distress (LEEP vs. surveillance in Model 3, B = −0.63, 95% CI −4.68 to +3.42; Table 3, Figure 2). With both HADS subscales entered together, depressive symptoms did not reach significance for the continuous outcome, although the point estimate remained negative (HADS-D B = −0.37, 95% CI −0.76 to +0.02, *p* = 0.064), and anxiety was unrelated (HADS-A *p* = 0.82). The strongest correlate throughout was marital status, with married women scoring higher than unmarried women (Figure 2).

For the clinically interpretable binary outcome, higher depressive symptom burden was associated with sexual dysfunction: in the adjusted logistic model each additional HADS-D point increased the odds of FSFI-defined dysfunction (OR = 1.14, 95% CI 1.03–1.26, *p* = 0.009), whereas no statistically significant associations were observed for anxiety or the management group (Table 4). Threshold-based (dichotomous) analyses of HADS-D were exploratory and are reported in the Supplementary Materials.

### 3.4. Sensitivity and Correlation Analyses

Sensitivity analyses did not materially alter the primary findings (Supplementary Table S4). When the composite severity score replaced the management group, no statistically significant association was observed between severity and FSFI, and the HADS-D association remained similar; restricting the analysis to women who remained HPV-positive did not change the results, and excluding influential observations exceeding the conventional Cook’s-distance threshold did not materially change the findings. In an additional sensitivity analysis restricted to married women (n = 181), management pathway was not statistically associated with FSFI total score (colposcopy vs. surveillance: B = −0.15, 95% CI −3.02 to 2.71; LEEP vs. surveillance: B = −2.93, 95% CI −7.04 to 1.17). In a BMI-adjusted sensitivity analysis restricted to participants with complete BMI and covariate data (n = 132), management pathway was likewise not statistically associated with FSFI total score (LEEP vs. surveillance: B = −3.25, 95% CI −11.49 to 4.98, *p* = 0.439), and BMI itself was not significantly associated with FSFI total score (B = 0.17, 95% CI −0.20 to 0.54, *p* = 0.363), with imprecise estimates owing to the reduced sample size (Supplementary Table S4). In exploratory correlation analysis, FSFI correlated more strongly with HADS-D (ρ = −0.22) than with HADS-A (ρ = −0.13), and depressive symptoms correlated moderately and negatively with all four WHOQOL domains; all pairwise correlations remained significant after Benjamini–Hochberg correction. Full diagnostics, the correlation matrix, and the sensitivity analyses are provided in the Supplementary Materials.

## 4. Discussion

In this cross-sectional cohort of reproductive-age women with a history of hrHPV positivity, sexual function, quality of life, and mood showed no statistically significant differences across the three management steps—surveillance, colposcopy, and LEEP. Although clinical severity differed substantially between the groups, patient-reported outcomes showed no statistically significant differences. No statistically significant association between management pathway and sexual function was observed after multivariable adjustment. Instead, depressive symptom burden showed the most consistent association with sexual dysfunction, whereas anxiety did not, and marital status was the strongest overall correlate.

The LEEP group carried the heaviest objective disease burden, with nearly four-fifths showing CIN2+ on biopsy, yet reported sexual function, anxiety, depression, and quality of life that did not differ significantly from women under surveillance. The absence of statistically significant between-group differences should not, however, be interpreted as evidence that LEEP has no psychosexual effect. Management pathway was closely linked to cervical disease severity and the indication for intervention, resulting in substantial confounding by indication. Although the sensitivity analysis replacing management group with the composite cervical-abnormality severity score yielded similar findings, residual confounding cannot be excluded; our results therefore indicate that the management pathway was not statistically associated with sexual function at the time of assessment, rather than demonstrating the absence of an independent effect of the procedure. Moreover, the study was powered to detect a medium between-group effect (Cohen’s f ≈ 0.25) rather than designed around a predefined equivalence margin; the null findings therefore do not establish equivalence or non-inferiority, and the confidence interval for the adjusted LEEP-versus-surveillance estimate (B = −0.63, 95% CI −4.68 to +3.42) remains compatible with potentially meaningful differences in FSFI total score. No validated MCID for the FSFI total score has been established specifically for women with hrHPV positivity or cervical dysplasia. As a distribution-based reference [21], one-half of the observed FSFI standard deviation in our cohort was approximately 4.9 points. The lower confidence limit for the adjusted LEEP-versus-surveillance estimate (−4.68 points, approximately 0.48 SD) approached this magnitude; therefore, although no statistically significant association was observed, a potentially important adverse difference cannot be excluded. This distribution-based reference should not be interpreted as a validated clinical threshold. These observations help reconcile a divided literature. Some studies report lower FSFI scores after colposcopy and LEEP [6,7], whereas several prospective studies find no significant change—or even improvement—after treatment [8,9,10]. Collectively, these studies suggest that persistent psychosexual difficulty may reflect psychological and biological factors in addition to any effects related to cervical excision. Our cross-sectional comparison of all three steps, with distress measured directly, is consistent with that interpretation: the management pathway was not statistically associated with sexual function after multivariable adjustment.

That depressive symptoms, rather than anxiety, carried the more consistent association merits comment. Anxiety in this setting may reflect carcinophobia and the situational uncertainty surrounding cervical assessment and the diagnosis-to-treatment interval, and may therefore fluctuate, diminishing as diagnostic uncertainty decreases, whereas depressive symptoms may have broader and more enduring associations with sexual desire, motivation, and pleasure. This accords with prior work in which anxiety and depressive symptoms, rather than sexual or procedural variables, showed the largest associations with impaired quality of life in HPV-positive women [5,12,13]. The relevance of psychological burden is not confined to HPV: in patients with other sexually transmitted infections, depressive symptoms have been reported to be more common and severe, and sexual functioning poorer, than in a non-STI comparison group, underscoring the broader psychosexual impact of these conditions [22]. We interpret the finding cautiously: it was significant for the binary FSFI-defined dysfunction outcome but not for the continuous total score, and HADS is a screening rather than a diagnostic instrument. Moreover, because depressive symptoms and sexual function were assessed concurrently, the cross-sectional design cannot establish the direction of this association: depressive symptoms may contribute to sexual difficulties, reduced sexual function may exacerbate depressive symptoms, or both may arise from shared psychosocial factors. These findings should therefore be regarded as exploratory and hypothesis-generating rather than as evidence that depressive symptoms are an established determinant of sexual dysfunction.

Marital status showed the largest adjusted association with FSFI total score in the primary models, highlighting the importance of relational context when interpreting sexual function. However, marital status is only a crude proxy for this context and does not capture current partnership characteristics, relationship duration or quality, partner-related factors, or other interpersonal influences; this finding should therefore not be interpreted as evidence of a specific relational mechanism. Taken together, the findings suggest that psychosocial and relational context may be important when interpreting sexual function in women with a history of hrHPV positivity, while the present cross-sectional data do not establish that these factors are more causally important than the management pathway.

These observations support considering sexual function within a broader psychosocial and relational assessment rather than interpreting it solely in relation to the cervical management pathway, and they do not suggest that psychosexual concerns are confined to women undergoing excisional treatment. However, because the study is cross-sectional and several relevant relational and psychosocial factors were not directly measured, these observations should be regarded as hypothesis-generating rather than as a basis for specific clinical management recommendations, and warrant confirmation in prospective, controlled studies.

Strengths of this study include a relatively large sample, the simultaneous comparison of all three management steps, the combined use of validated instruments, and a conservative analytic approach with robust standard errors, pre-specified severity handling, and multiple sensitivity analyses. Several limitations should be acknowledged. The cross-sectional design precludes causal inference and cannot establish whether colposcopy or LEEP itself affects sexual function, quality of life, or psychological distress. In addition, management step and disease severity are structurally confounded, so the results reflect the management pathway together with disease severity rather than an isolated effect of LEEP, and substantial residual confounding by indication cannot be excluded. The study was conducted at a single center. In addition, FSFI scores are inherently influenced by relationship and sexual-activity context. Several potentially relevant determinants of female sexual function were not available in our dataset, including hormonal contraceptive use, relationship duration and quality, partner-related factors, intercourse frequency, previous sexual dysfunction, fertility concerns, and time since HPV diagnosis; therefore, residual confounding cannot be excluded. Body mass index, which differed across management groups, was available for approximately half of the cohort and was not included in the primary models. A BMI-adjusted sensitivity analysis yielded no statistically significant association between management pathway and FSFI, although the substantially reduced sample resulted in wide confidence intervals. HADS measures symptom severity, not clinical diagnosis, and the depression–FSFI association did not reach significance for the continuous outcome. In addition, we did not administer instruments specific to cancer-related fear (carcinophobia) or HPV-related stigma; the HADS captures general anxiety and depressive symptoms rather than these disease-specific dimensions, which may therefore be under-represented in our assessment of the psychosocial burden. Future studies should evaluate these constructs using disease-specific instruments. The interval between colposcopy or LEEP and questionnaire administration was not recorded; therefore, short-term procedural effects, subsequent recovery over time, and pre-existing differences in patient-reported outcomes could not be distinguished. In the absence of pre-procedure (baseline) assessment and longitudinal follow-up, within-patient changes attributable to the procedure itself could not be determined. In addition, participants may have been assessed at different stages of post-procedural recovery; pooling observations obtained at potentially very different time points may have attenuated transient or persistent time-dependent effects and could obscure genuine differences between management pathways. There was no HPV-negative or general-population comparison group. Consequently, although FSFI-defined sexual dysfunction was common in our cohort (approximately 60%), this prevalence cannot be attributed to HPV-related disease and may, at least in part, reflect the background prevalence of sexual dysfunction in comparable populations. The proportion of sexual dysfunction attributable to HPV positivity itself therefore cannot be determined from these data. Because eligibility was restricted to women who had been sexually active during the preceding four weeks, women who had discontinued sexual activity because of HPV-related anxiety, stigma, pain, relationship difficulties, treatment-related concerns, or sexual avoidance were not captured. Although this criterion was necessary to permit interpretable FSFI assessment, it may have preferentially excluded women with the greatest sexual impairment and may therefore have led to underestimation of the overall psychosexual burden. The exclusion of women with active psychiatric disorders may have further reduced the observed level of psychological distress and limits the generalizability of the HADS findings. Our estimates may therefore underestimate the overall psychosexual burden. Moreover, if such exclusion was more frequent in groups with greater disease or treatment burden, it could also have attenuated underlying between-group differences in sexual function. Finally, individual non-16/18 hrHPV genotypes within the pooled assay channel could not be distinguished.

## 5. Conclusions

Among reproductive-age women with a history of hrHPV positivity, sexual function, quality of life, and mood showed no statistically significant differences across surveillance, colposcopy, and LEEP, and no statistically significant association between management pathway and sexual function was observed after adjustment. FSFI-defined sexual dysfunction was frequent across all three management groups; however, because the study lacked an HPV-negative or general-population comparison group, this prevalence should be interpreted descriptively rather than as evidence of an HPV-associated burden. In these exploratory analyses, depressive symptoms showed the most consistent association with sexual dysfunction, particularly for the binary FSFI-defined outcome, although their association with the continuous FSFI total score did not reach statistical significance and the cross-sectional design precludes any determination of the direction of this relationship. Relational and psychosocial context may be important when interpreting sexual function, although marital status is only a crude proxy for this context; these findings therefore highlight the potential importance of assessing psychosocial and relational factors, alongside psychological distress, within the broader psychosexual care of women with a history of hrHPV positivity. Because the design is cross-sectional and several relational and psychosocial factors were not directly measured, these observations are hypothesis-generating, and prospective studies incorporating more detailed measures of relationship context and HPV-specific distress are needed before specific clinical recommendations can be made.

## Figures and Tables

**Figure 1:**
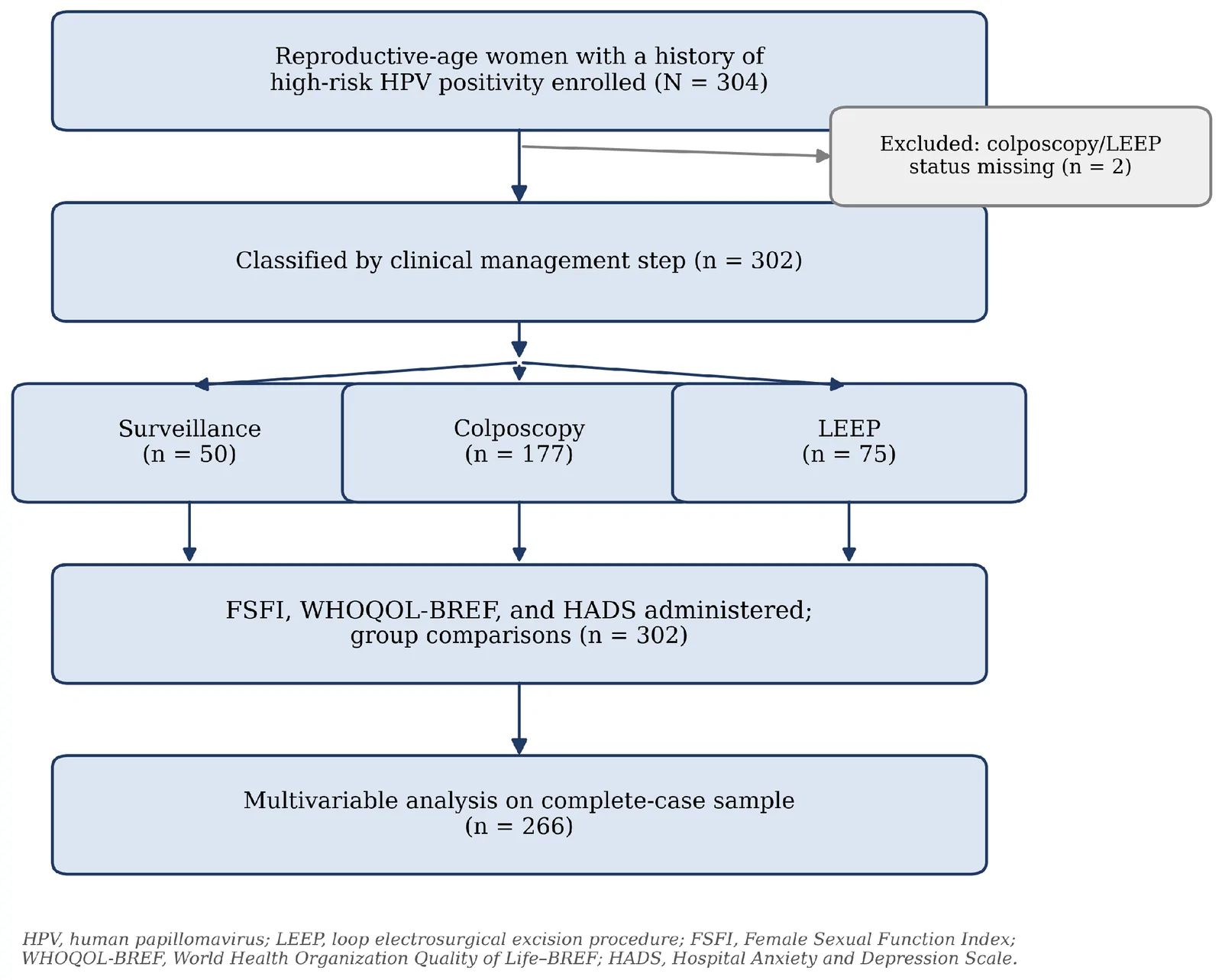
Participant flow through the study.

**Figure 2:**
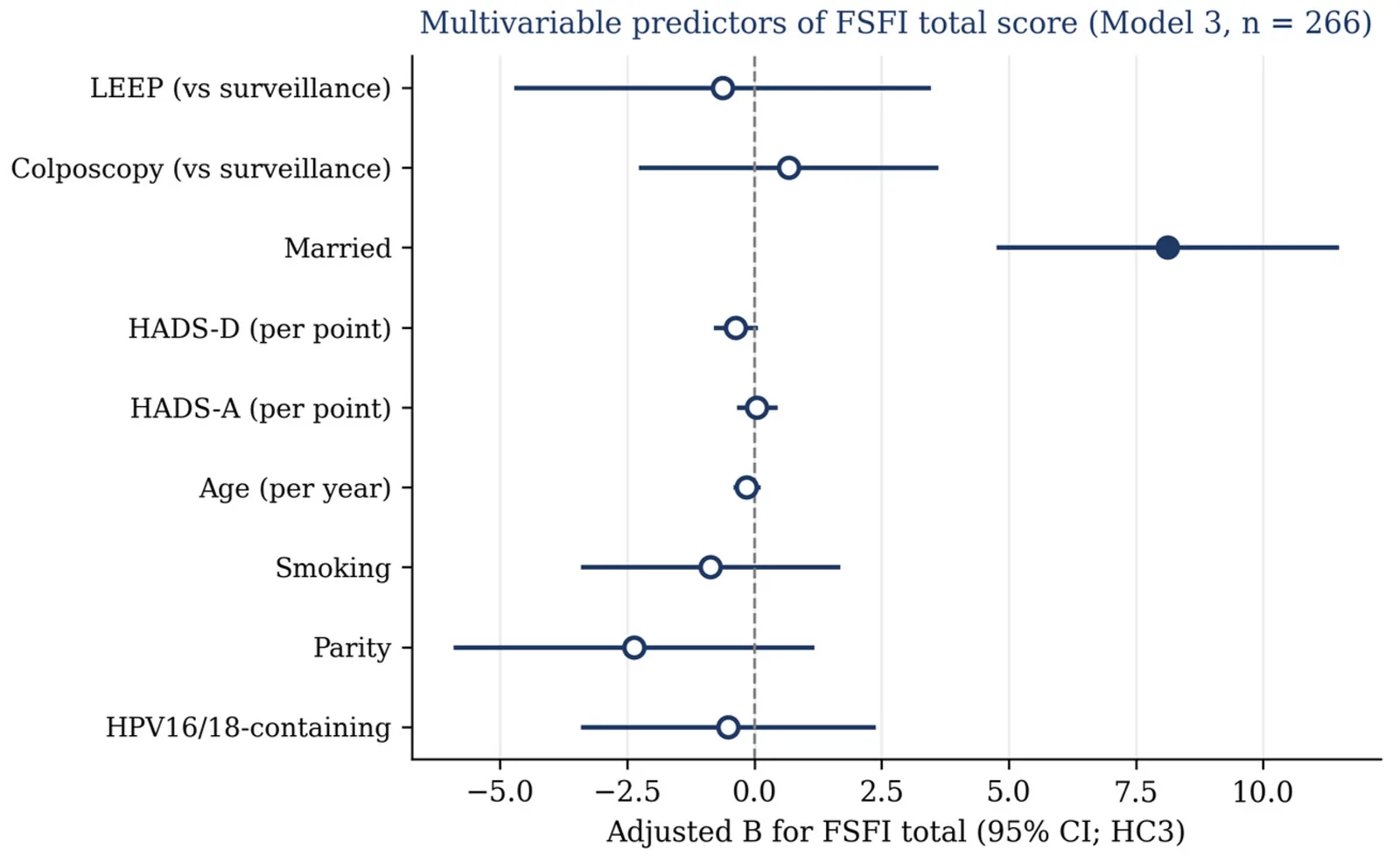
Adjusted associations with FSFI total score (Model 3). Filled markers indicate *p* < 0.05; the dashed line marks the null.

**Table 1:** Sociodemographic and clinical characteristics by management group.

Variable	Surveillance (n = 50)	Colposcopy (n = 177)	LEEP (n = 75)	*p*
Age (years)	37.0 [29.0–42.8]	37.0 [32.0–42.0]	38.0 [32.5–42.0]	0.545
BMI (kg/m^2^) ^b^	30.5 [26.2–34.1]	24.2 [22.4–27.6]	24.2 [22.9–28.2]	0.005
Marital status				0.045
Married	42 (84.0)	114 (64.4)	50 (66.7)	
Widowed	3 (6.0)	29 (16.4)	16 (21.3)	
Single	5 (10.0)	34 (19.2)	9 (12.0)	
Education ^a^				0.767
Primary	21 (43.8)	57 (32.9)	24 (33.3)	
High school	12 (25.0)	53 (30.6)	22 (30.6)	
University	2 (4.2)	11 (6.4)	8 (11.1)	
Postgraduate	13 (27.1)	51 (29.5)	18 (25.0)	
Smoking				0.653
No	32 (64.0)	93 (56.7)	38 (57.6)	
Yes	18 (36.0)	71 (43.3)	28 (42.4)	
Parity				0.293
Nulliparous	13 (26.5)	60 (35.1)	19 (26.4)	
Parous	36 (73.5)	111 (64.9)	53 (73.6)	
HPV genotype ^a^				<0.001
Currently hrHPV-negative	18 (36.0)	14 (7.9)	2 (2.7)	
HPV16	0 (0.0)	37 (20.9)	27 (36.0)	
HPV18	0 (0.0)	6 (3.4)	6 (8.0)	
Isolated other hrHPV	32 (64.0)	84 (47.5)	20 (26.7)	
Multiple (16/18-containing)	0 (0.0)	36 (20.3)	20 (26.7)	
Cytology ^a^				<0.001
Normal	31 (62.0)	46 (26.0)	11 (14.7)	
ASC-US	19 (38.0)	71 (40.1)	30 (40.0)	
LSIL	0 (0.0)	54 (30.5)	20 (26.7)	
ASC-H	0 (0.0)	3 (1.7)	5 (6.7)	
HSIL	0 (0.0)	1 (0.6)	9 (12.0)	
AGC/AGUS	0 (0.0)	2 (1.1)	0 (0.0)	
Biopsy ^a^				<0.001
No biopsy	50 (100.0)	51 (28.8)	4 (5.3)	
Normal	0 (0.0)	63 (35.6)	2 (2.7)	
CIN1	0 (0.0)	62 (35.0)	10 (13.3)	
CIN2	0 (0.0)	1 (0.6)	36 (48.0)	
CIN3	0 (0.0)	0 (0.0)	23 (30.7)	

Continuous variables are median [IQR]; categorical variables n (%). Percentages are based on available observations; denominators may vary owing to missing data. ^a^ Monte Carlo approximation of the Fisher–Freeman–Halton exact test owing to sparse expected cell counts. ^b^ BMI was available in ~50% of cases (available-case only) and was not entered into the primary multivariable models; it was evaluated separately in Sensitivity D.

**Table 2:** Patient-reported outcomes by management group.

Outcome	Surveillance	Colposcopy	LEEP	*p*	ε^2^
FSFI total	23.8 [20.4–28.3]	25.2 [16.9–28.9]	24.5 [17.5–28.6]	0.897	0.000
Desire	3.6 [3.0–4.2]	3.6 [3.0–4.2]	3.6 [2.4–4.8]	0.648	0.000
Arousal	4.0 [3.0–4.8]	3.9 [2.4–4.8]	3.3 [2.4–4.5]	0.279	0.002
Lubrication	3.9 [3.3–4.8]	4.2 [3.3–4.8]	4.2 [3.3–4.9]	0.803	0.000
Orgasm	4.4 [3.6–5.2]	4.4 [2.8–5.2]	4.2 [2.4–5.2]	0.648	0.000
Satisfaction	4.8 [3.6–5.6]	4.8 [2.4–5.6]	4.4 [2.5–5.6]	0.556	0.000
Pain	4.0 [2.4–4.8]	4.4 [1.6–5.8]	4.2 [1.7–5.9]	0.649	0.000
HADS-A	8.5 [6.0–11.8]	9.0 [6.0–12.0]	8.0 [6.0–11.0]	0.690	0.000
HADS-D	6.5 [3.0–8.8]	6.0 [4.0–9.0]	5.0 [4.0–8.0]	0.338	0.001
WHOQOL Physical	53.6 [46.4–64.3]	53.6 [48.2–60.7]	57.7 [50.0–67.9]	0.090	0.010
WHOQOL Psychological	66.7 [54.2–75.0]	70.8 [58.3–75.0]	70.8 [62.5–79.2]	0.206	0.004
WHOQOL Social	66.7 [50.0–83.3]	66.7 [50.0–75.0]	75.0 [58.3–83.3]	0.061	0.013
WHOQOL Environment	68.8 [59.4–81.2]	65.6 [56.2–78.1]	68.8 [59.4–80.5]	0.286	0.002
FSD < 26.55, n/N (%)	33/49 (67.3)	104/175 (59.4)	44/74 (59.5)	0.584	—

Values are median [IQR]. FSFI subdomains are weighted scores; higher scores indicate better function. WHOQOL domains are 0–100. Negative ε^2^ estimates were truncated at zero.

**Table 3:** Multivariable linear regression for FSFI total score (B, 95% CI; HC3-robust SE).

Variable	Model 1	Model 2	Model 3
Colposcopy (vs. surveillance)	−1.54 (−4.32, +1.25)	+0.62 (−2.28, +3.51)	+0.67 (−2.23, +3.56)
LEEP (vs. surveillance)	−2.65 (−6.09, +0.79)	−0.45 (−4.51, +3.60)	−0.63 (−4.68, +3.42)
Age	—	−0.15 (−0.37, +0.07)	−0.15 (−0.37, +0.07)
Married (vs. not married)	—	+8.25 (+5.03, +11.46) *	+8.12 (+4.80, +11.44) *
High school (vs. primary)	—	+1.29 (−1.69, +4.28)	+0.77 (−2.25, +3.79)
University (vs. primary)	—	+2.41 (−2.15, +6.98)	+1.77 (−2.80, +6.35)
Postgraduate (vs. primary)	—	+1.19 (−2.16, +4.53)	+0.77 (−2.63, +4.18)
Smoking (yes vs. no)	—	−1.02 (−3.49, +1.45)	−0.86 (−3.37, +1.64)
Parity (parous vs. nulliparous)	—	−2.52 (−6.04, +1.00)	−2.37 (−5.87, +1.12)
HPV16/18-containing (vs. isolated other hrHPV)	—	−0.64 (−3.50, +2.22)	−0.52 (−3.38, +2.33)
Currently hrHPV-negative (vs. isolated other hrHPV)	—	+1.72 (−1.59, +5.02)	+1.74 (−1.56, +5.04)
HADS-A	—	—	+0.05 (−0.30, +0.40)
HADS-D	—	—	−0.37 (−0.76, +0.02)
Adjusted R^2^	0.000	0.131	0.141
n	266	266	266

* *p* < 0.05. Reference group: surveillance; HPV reference: isolated other hrHPV. All three models were fitted on the same complete-case sample (n = 266).

**Table 4:** Logistic regression for FSFI-defined sexual dysfunction (FSFI < 26.55).

Variable	OR (95% CI)	*p*
Colposcopy (vs. surveillance)	0.61 (0.27–1.39)	0.236
LEEP (vs. surveillance)	0.77 (0.29–2.09)	0.611
Age	1.04 (0.99–1.09)	0.120
Married (vs. not married)	0.35 (0.18–0.70)	0.003 *
High school (vs. primary)	0.74 (0.37–1.51)	0.413
University (vs. primary)	0.61 (0.19–1.94)	0.406
Postgraduate (vs. primary)	0.48 (0.22–1.04)	0.064
Smoking (yes vs. no)	1.51 (0.86–2.63)	0.149
Parity (parous vs. nulliparous)	1.25 (0.57–2.75)	0.573
HPV16/18-containing (vs. isolated other hrHPV)	1.00 (0.54–1.86)	0.999
Currently hrHPV-negative (vs. isolated other hrHPV)	1.27 (0.51–3.20)	0.607
HADS-A	0.98 (0.91–1.07)	0.674
HADS-D	1.14 (1.03–1.26)	0.009 *

* *p* < 0.05. n = 266. Reference group: surveillance.

## Data Availability

The data are not publicly available because they contain sensitive personal and sexual-health information; de-identified data may be available from the corresponding author upon reasonable request and subject to institutional approval.

## Supplementary Materials

The following supporting information can be downloaded at https://www.mdpi.com/article/10.3390/jcm15197386/s1: Table S1. missing-data summary; Table S2. partial-R^2^ and standardized-coefficient estimates; Table S3. regression diagnostics; Table S4. additional sensitivity analyses; Table S5. logistic-model performance; Table S6. FSFI between-group mean differences; Figure S1. FSFI–HADS–WHOQOL correlation matrix; STROBE checklist for cross-sectional studies.

## Abbreviations

The following abbreviations are used in this manuscript:

HPV	human papillomavirus
hrHPV	high-risk human papillomavirus
LEEP	loop electrosurgical excision procedure
CIN	cervical intraepithelial neoplasia
ASC-US	atypical squamous cells of undetermined significance
LSIL	low-grade squamous intraepithelial lesion
ASC-H	atypical squamous cells, cannot exclude HSIL
HSIL	high-grade squamous intraepithelial lesion
AGC	atypical glandular cells
AGUS	atypical glandular cells of undetermined significance
FSFI	Female Sexual Function Index
WHOQOL-BREF	World Health Organization Quality of Life, Brief version
HADS	Hospital Anxiety and Depression Scale
HADS-A	Hospital Anxiety and Depression Scale, anxiety subscale
HADS-D	Hospital Anxiety and Depression Scale, depression subscale
BMI	body mass index
STROBE	Strengthening the Reporting of Observational Studies in Epidemiology
HC3	heteroscedasticity-consistent standard errors (type 3)
OR	odds ratio
CI	confidence interval
IQR	interquartile range
