# Supplementary material for: Sexual Function and Psychological Distress Across Surveillance, Colposcopy, and LEEP in Women with a History of High-Risk HPV Positivity: A Cross-Sectional Study
Source: J Clin Med. 2026 Sep 23;15(19):7386. doi: 10.3390/jcm15197386 (PMC13666160; doi:10.3390/jcm15197386)
Supplement: Supplementary file 1 [file jcm-15-07386-s001.zip › jcm-4535798-supplementary.pdf]

Supplementary Statistical Analyses

This supplement accompanies the main results, in the order cited in the manuscript: missing data (Table S1); partial R^2 and selected standardized coefficients for continuous predictors (Table S2); regression diagnostics (Table S3); additional sensitivity analyses (Table S4); logistic-model performance (Table S5); FSFI between-group mean differences (Table S6); and the FSFI–HADS–WHOQOL correlation matrix (Figure S1).

Table S1. Missing data and complete-case sample

Variable	Missing n (%)
FSFI total	4 (1.3)
HADS-A	0 (0.0)
HADS-D	0 (0.0)
WHOQOL (any domain)	8 (2.6)
Age	0 (0.0)
Marital status	0 (0.0)
Education	10 (3.3)
Smoking	22 (7.3)
Parity	10 (3.3)
HPV genotype	0 (0.0)
BMI	153 (50.7)

Missing HADS values were completed from source forms, so HADS-A and HADS-D are now 100% complete; missingness in the primary outcome (FSFI) is also low (4 (1.3)). Because Model 3 is complete-case, cases with any missing model variable are excluded. The complete-case sample is 266/302 (~12% lost); this loss is now driven by covariate missingness rather than HADS (smoking 22, parity 10, education 10, FSFI 4). All three regression models (Models 1–3) were fitted on the same complete-case sample (n=266), so coefficient changes and R^2 differences reflect only the added covariates; the crude group comparison on all participants is given in Table 2. Given the low missingness in the primary outcome and the extensive missingness in BMI, which was therefore excluded from the primary multivariable models and evaluated separately in Sensitivity D, the primary analyses used complete cases; multiple imputation was not performed.

Table S2. Partial R^2 and standardized coefficients (Model 3)

Variable	Partial R^2	p
Colposcopy (vs surveillance)	0.1%	0.651
LEEP (vs surveillance)	0.0%	0.761
Age	1.0%	0.171
Married (vs not married)	11.9%	<0.001
High school (vs primary)	0.1%	0.616
University (vs primary)	0.2%	0.447
Postgraduate (vs primary)	0.1%	0.657
Smoking (yes vs no)	0.2%	0.500
Parity (parous vs nulliparous)	0.8%	0.183
HPV16/18-containing	0.1%	0.719
Currently hrHPV-negative	0.3%	0.301
HADS-A	0.0%	0.781
HADS-D	1.3%	0.064

Partial R^2 is reported for all predictors (comparable across continuous and categorical terms); standardized β are given only for the continuous predictors, since standardized coefficients for dummy variables are not directly comparable — for categorical predictors the unstandardized B with 95% CI in Table 3 should be used. The strongest correlate is marital status (partial $R^2 \approx 12\%$); in Model 3, married women scored approximately 8 FSFI points higher than unmarried women (Table 3). Among continuous predictors the standardized β were HADS-D -0.14, age -0.11, and HADS-A 0.02 (only HADS-D approached significance; HC3 p=0.064). Adding psychological distress increased the unadjusted explained variance from R^2=0.167 to 0.183 (ΔR^2=0.016); the corresponding adjusted R^2 values were 0.131 and 0.141 (Table 3). Partial R^2 values were derived from the ordinary least-squares model, whereas the p-values and confidence intervals reported here and in Table 3 were based on HC3 robust standard errors.

In the fully adjusted continuous model HADS-D did not reach statistical significance (B=-0.37, 95% CI -0.76 to 0.02, HC3 p=0.064). HADS-A and HADS-D were moderately correlated (ρ≈0.63), although VIF values did not indicate problematic multicollinearity; in a supporting model excluding HADS-A, HADS-D was associated with lower FSFI scores (B=-0.33, 95% CI -0.62 to -0.04, p=0.028), a post-hoc analysis that did not replace the pre-specified model including both HADS subscales. The association was clearer for the binary outcome (see Table 4 in the main manuscript) and for elevated depressive symptoms (see S4). Overall, depressive symptoms were the most consistent psychological correlate of sexual dysfunction—significant for the binary FSFI-defined outcome, though not for the continuous score—while the strongest overall correlate was marital status.

Table S3. Regression diagnostics (Model 3)

Variable	VIF
Age	1.46
Married	1.32
Smoking	1.08
Parity	1.92
HADS-A	1.84
HADS-D	1.87
HPV16/18-containing	1.36
Currently hrHPV-negative	1.21
Education (max across dummy terms)	1.81

All VIF values are <2 (maximum 1.92); VIF values did not indicate problematic multicollinearity, including when HADS-A and HADS-D were entered simultaneously, and education (entered as three dummy terms) shows a maximum VIF of 1.81. Because heteroscedasticity was detected (Breusch–Pagan p<0.001), HC3 heteroscedasticity-consistent standard errors were used for inference; residuals were also non-normal (Shapiro–Wilk p<0.001). On influence analysis, 18 observations exceeded the Cook's-distance threshold (4/n=0.015) but the maximum Cook's distance was 0.036 (≪1), so no single observation drove the model.

Table S4. Additional sensitivity analyses

Influence (Cook's distance): re-fitting Model 3 after excluding the 18 observations exceeding the conventional Cook's-distance threshold (n=248) did not alter the direction of the findings or reveal an association with management pathway (LEEP p=0.771). This is reported as a robustness check only; the primary estimate remains Model 3 fitted to the full complete-case sample, without influence-based exclusions.

Marital status (three categories): to confirm that binary 'married vs not' coding did not artificially inflate the estimate, marital status was modelled with three categories (reference: married). Relative to married women, both widowed (B=-8.11 (-12.3, -3.9), p<0.001) and single (B=-8.14 (-12.9, -3.4), p<0.001) women had substantially and almost identically lower FSFI, supporting the binary coding. The data capture marital status, not partner status, and results are described accordingly.

Elevated depressive symptoms (post-hoc): in a model replacing continuous HADS-D with a binary indicator of elevated depressive symptoms (HADS-D≥11), adjusted for the same covariates (n=266), women with elevated

depressive symptoms scored significantly lower on FSFI (adjusted B=-4.11, 95% CI -7.33 to -0.88, p=0.013). This analysis and the model excluding HADS-A were not pre-specified as primary and are reported as post-hoc / supporting analyses; HADS-D≥11 denotes elevated depressive symptoms on a screening scale, not a clinical diagnosis of depression.

HADS-D threshold sensitivity (exploratory): HADS-D was dichotomised at two thresholds—the commonly applied ≥11 (41/302 women) and the screening cut-off of ≥7 from the Turkish validation (133/302). Results differed according to the threshold used: the post-hoc association with FSFI was present with HADS-D≥11 (B=-4.11, p=0.013) but not with the lower ≥7 cut-off (B=-0.45, 95% CI -2.79 to 1.9, p=0.709; n=266). Because these analyses were exploratory and threshold-dependent, they were not used as the primary basis for inference; the continuous HADS-D analyses do not depend on a threshold.

Labelling notes: the protocol-specified cut-off FSFI <26.55 was retained for the primary analysis; no participant scored exactly 26.55, so <26.55 and ≤26.55 are numerically identical here. Sexual dysfunction is defined by the FSFI cut-off (a questionnaire-based threshold), not by clinical diagnosis. HPV genotyping used a single assay (cobas 4800 HPV Test) with three reporting channels (HPV16, HPV18, and a pooled panel of 12 other high-risk types); since positivity in ≥2 channels necessarily involves HPV16 and/or HPV18, the 'HPV16/18-containing' predictor captures isolated HPV16, isolated HPV18, and all multiple infections (n=132) versus isolated other-hrHPV infection (n=136), so HPV16/18 carriers are correctly classified rather than undercounted.

Cervical-abnormality severity replacing management pathway (Sensitivity A): because management pathway and cervical disease severity are structurally confounded, the primary FSFI Model 3 was refitted with the composite cervical-abnormality severity score in place of management group (n=266). The severity score was the maximum of the cytology-derived grade (0 = normal; 1 = ASC-US/LSIL; 2 = ASC-H/HSIL/AGC/AGUS) and the biopsy-derived grade (0 = normal; 1 = CIN1; 2 = CIN2/CIN3), with cytology determining the grade when biopsy was not performed. Cervical-abnormality severity was not statistically associated with FSFI total score (grade 1 vs 0 B=1.25, 95% CI -1.51 to 4.01; grade 2 vs 0 B=-1.35, 95% CI -5.17 to 2.47), and the association of depressive symptoms with FSFI was similar to the primary model (HADS-D B=-0.39, 95% CI -0.768 to -0.003, p=0.048).

Table S4a. Sensitivity A — cervical-abnormality severity replacing management pathway (n=266)

Covariate	B	95% CI	p
Severity grade 1 (vs grade 0)	1.25	-1.51 to 4.01	0.376
Severity grade 2 (vs grade 0)	-1.35	-5.17 to 2.47	0.488
Age	-0.16	-0.38 to 0.05	0.139
Married (vs not married)	8.09	4.78 to 11.40	<0.001
High school (vs primary)	0.68	-2.37 to 3.74	0.662
University (vs primary)	2.15	-2.47 to 6.77	0.362
Postgraduate (vs primary)	0.79	-2.61 to 4.19	0.649
Smoking (yes vs no)	-0.82	-3.33 to 1.68	0.518
Parity (parous vs nulliparous)	-2.22	-5.75 to 1.30	0.216
HPV16/18-containing (vs isolated other hrHPV)	-0.24	-2.91 to 2.43	0.860
Currently hrHPV-negative (vs isolated other hrHPV)	1.38	-1.84 to 4.60	0.401
HADS-A	0.04	-0.30 to 0.39	0.812
HADS-D	-0.39	-0.768 to -0.003	0.048

Multivariable linear regression for FSFI total score; HC3 robust standard errors; reference: severity grade 0. B, unstandardised regression coefficient. Covariate labels match Table 3 of the main manuscript.

Currently hrHPV-positive subgroup (Sensitivity B): the primary FSFI Model 3 was refitted after restricting the sample to women who remained hrHPV-positive at the study visit (n=235), excluding those with a documented prior positive result who were currently hrHPV-negative. Management pathway remained not statistically associated with FSFI total score (colposcopy vs surveillance B=1.31, 95% CI -2.06 to 4.67; LEEP vs surveillance

B=0.02, 95% CI -4.57 to 4.60), and the association of depressive symptoms with FSFI was similar to the full-cohort model (HADS-D B=-0.40, 95% CI -0.82 to 0.01, p=0.058).

Table S4b. Sensitivity B — currently hrHPV-positive subgroup (n=235)

Covariate	B	95% CI	p
Colposcopy (vs surveillance)	1.31	-2.06 to 4.67	0.447
LEEP (vs surveillance)	0.02	-4.57 to 4.60	0.994
Age	-0.14	-0.39 to 0.10	0.250
Married (vs not married)	8.82	5.34 to 12.31	<0.001
High school (vs primary)	0.98	-2.34 to 4.31	0.563
University (vs primary)	1.66	-3.24 to 6.55	0.508
Postgraduate (vs primary)	0.35	-3.41 to 4.10	0.857
Smoking (yes vs no)	-1.26	-4.00 to 1.49	0.369
Parity (parous vs nulliparous)	-2.21	-6.11 to 1.68	0.265
HPV16/18-containing (vs isolated other hrHPV)	-0.63	-3.58 to 2.31	0.673
HADS-A	0.12	-0.27 to 0.51	0.552
HADS-D	-0.40	-0.82 to 0.01	0.058

Multivariable linear regression for FSFI total score; HC3 robust standard errors; reference group: surveillance; B, unstandardised regression coefficient. Covariate labels match Table 3 of the main manuscript.

Married women only (Sensitivity C): the primary FSFI Model 3 was refitted after restricting the sample to married women (n=181), with marital status omitted from the model. Management pathway remained not statistically associated with FSFI total score (colposcopy vs surveillance B=-0.15, 95% CI -3.02 to 2.71; LEEP vs surveillance B=-2.93, 95% CI -7.04 to 1.17). The LEEP point estimate was more negative than in the full cohort, but the confidence interval was wide and did not exclude a potentially meaningful difference. The association of depressive symptoms with FSFI was somewhat stronger in this subgroup (HADS-D B=-0.43, 95% CI -0.84 to -0.02, p=0.038).

Table S4c. Sensitivity C — married women only (n=181)

Covariate	B	95% CI	p
Colposcopy (vs surveillance)	-0.15	-3.02 to 2.71	0.917
LEEP (vs surveillance)	-2.93	-7.04 to 1.17	0.162
Age	-0.21	-0.44 to 0.02	0.075
High school (vs primary)	-0.66	-3.87 to 2.55	0.686
University (vs primary)	2.44	-1.73 to 6.62	0.251
Postgraduate (vs primary)	1.58	-1.74 to 4.90	0.350
Smoking (yes vs no)	-0.83	-3.33 to 1.68	0.518
Parity (parous vs nulliparous)	-0.91	-4.32 to 2.49	0.599
HPV16/18-containing (vs isolated other hrHPV)	-0.36	-3.19 to 2.46	0.802
Currently hrHPV-negative (vs isolated other hrHPV)	-1.17	-4.49 to 2.16	0.492
HADS-A	-0.01	-0.39 to 0.36	0.939
HADS-D	-0.43	-0.84 to -0.02	0.038

Multivariable linear regression for FSFI total score; HC3 robust standard errors; reference group: surveillance; B, unstandardised regression coefficient. Covariate labels match Table 3 of the main manuscript.

BMI-adjusted (Sensitivity D): body mass index was available for 149 of 302 group-assigned participants (49.3%) and differed across management groups; it was therefore not included in the primary models. In a sensitivity analysis adding BMI among participants with complete BMI and covariate data (n=132), management pathway

remained not statistically associated with FSFI total score (LEEP vs surveillance B=-3.25, 95% CI -11.49 to 4.98, p=0.439), and BMI itself was not significantly associated with FSFI (B=0.17, 95% CI -0.20 to 0.54, p=0.363). Estimates were imprecise owing to the substantially reduced sample size, and the analysis does not exclude a potentially important difference.

Table S4d. Sensitivity D — BMI-adjusted, complete-case (n=132)

Covariate	B	95% CI	p
Colposcopy (vs surveillance)	-0.91	-8.04 to 6.22	0.803
LEEP (vs surveillance)	-3.25	-11.49 to 4.98	0.439
Age	-0.27	-0.62 to 0.08	0.130
Married (vs not married)	5.50	0.05 to 10.95	0.048
High school (vs primary)	0.21	-5.35 to 5.77	0.941
University (vs primary)	-1.94	-13.00 to 9.12	0.731
Postgraduate (vs primary)	-1.41	-7.66 to 4.84	0.658
Smoking (yes vs no)	-1.18	-5.28 to 2.92	0.572
Parity (parous vs nulliparous)	-0.00	-5.57 to 5.57	0.999
HPV16/18-containing (vs isolated other hrHPV)	2.94	-1.60 to 7.48	0.204
Currently hrHPV-negative (vs isolated other hrHPV)	3.05	-3.12 to 9.23	0.332
HADS-A	-0.13	-0.70 to 0.45	0.667
HADS-D	-0.57	-1.28 to 0.13	0.112
Body mass index (kg/m²)	0.17	-0.20 to 0.54	0.363

Multivariable linear regression for FSFI total score; HC3 robust standard errors; reference group: surveillance; B, unstandardised regression coefficient. Covariate labels match Table 3 of the main manuscript.

Table S5. Logistic model performance (FSD <26.55)

The secondary FSFI-dysfunction logistic model (fitted by maximum likelihood) showed acceptable discrimination (area under the ROC curve, AUC=0.709). As this model is a secondary, clinically oriented analysis intended to complement the continuous primary outcome, discrimination is reported for completeness.

	FSFI	HADS-A	HADS-D	WHO-Phys	WHO-Psych	WHO-Soc	WHO-Env
FSFI	1	-0.13*	-0.22***	0.18**	0.17**	0.49***	0.29***
HADS-A	-0.13*	1	0.63***	-0.35***	-0.39***	-0.30***	-0.40***
HADS-D	-0.22***	0.63***	1	-0.42***	-0.46***	-0.40***	-0.46***
WHO-Phys	0.18**	-0.35***	-0.42***	1	0.56***	0.44***	0.60***
WHO-Psych	0.17**	-0.39***	-0.46***	0.56***	1	0.49***	0.61***
WHO-Soc	0.49***	-0.30***	-0.40***	0.44***	0.49***	1	0.60***
WHO-Env	0.29***	-0.40***	-0.46***	0.60***	0.61***	0.60***	1

** p<0.05, ** p<0.01, *** p<0.001. Correlation analyses were exploratory; the stars show unadjusted p-values, but all pairwise associations remained significant after Benjamini–Hochberg FDR correction (largest adjusted p=0.027), including FSFI–HADS-D.*

Table S6. FSFI between-group mean differences with 95% CI

Comparison	Mean difference (95% CI)
Colposcopy − Surveillance	-1.19 (-3.81, +1.43)
LEEP − Surveillance	-1.71 (-4.87, +1.45)
LEEP − Colposcopy	-0.52 (-3.28, +2.24)

Mean differences and 95% CIs were computed using unpooled (Welch-type) standard errors with a normal approximation. Point estimates for between-group FSFI mean differences were small and all 95% CIs included zero; however, the confidence intervals were sufficiently wide that potentially important between-group differences cannot be excluded, and the findings should therefore be described as an absence of a statistically significant difference rather than as evidence of no difference.

Figure S1. Correlation matrix (Spearman): FSFI – HADS – WHOQOL-BREF

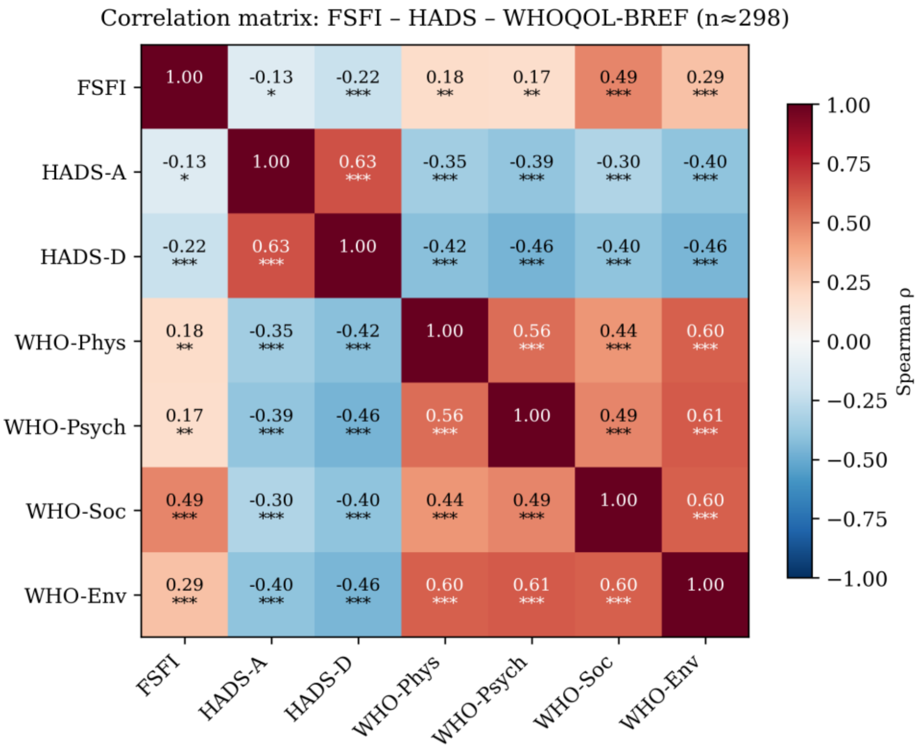

Among the psychological measures assessed, HADS-D showed the stronger correlation with FSFI ($\rho=-0.22$, $p<0.001$) than HADS-A ($\rho=-0.13$, $p=0.027$). Depressive symptoms also correlate moderately and negatively with all four WHOQOL domains ($\rho \approx -0.4$ to -0.5), consistent with a broad footprint of distress on both sexual function and quality of life.

Interpretive note: why depression is more consistent than anxiety

This is a Discussion (literature) point rather than a statistical one. That depressive symptoms carry the more consistent association when both HADS subscales are modelled together is compatible with anxiety being more closely linked to the situational uncertainty surrounding cervical assessment, whereas depressive symptoms may have broader associations with sexual desire, motivation, and pleasure. (Note: HADS-A is not a state-anxiety measure such as the STAI-State, so a strict state/trait dichotomy should be avoided.) This pattern is consistent with Uner & Korukcu, the Ilic series, and Calvo-Torres (larger effect sizes for anxiety/depression than for sexual factors). The emphasis should remain that depressive symptoms are the most consistent psychological correlate of sexual dysfunction — not that depression is its determinant.

Note on BMI

BMI was missing in ~50% of cases (shown as available-case only in Table 1; the associated p should be interpreted with caution). Owing to this high missingness, BMI was not included in the primary multivariable models; instead, it was evaluated in a dedicated sensitivity analysis (Sensitivity D; see Table S4d), which did not yield a statistically significant association between management pathway and FSFI, although estimates were imprecise because of the reduced sample size. In Table 1, BMI is flagged as available-case only (~50% missing).
